# The DNA barcode identification of *Dalbergia odorifera* T. Chen and *Dalbergia tonkinensis* Prain

**DOI:** 10.1186/s12870-023-04513-3

**Published:** 2023-11-07

**Authors:** Weijie Wang, Baixu Chen, Ruoke Ma, Mengji Qiao, Yunlin Fu

**Affiliations:** Guangxi Key Laboratory of Forest Ecology and Conservation, Key Laboratory of National Forestry and Grassland Administration on Cultivation of Fast-Growing Timber in Central South China, College of Forestry, Guangxi University, Nanning, 530004 China

**Keywords:** *Dalbergia odorifera*, *Dalbergia tonkinensis*, DNA barcoding, Identification, *trnH-psbA*

## Abstract

**Background:**

*Dalbergia odorifera* is a precious tree species with unique economic and medicinal values, which is difficult to distinguish from *Dalbergia tonkinensis* by traditional identification methods such as morphological characteristics and wood structure characteristics. It has been demonstrated that the identification of tree species can be effectively achieved using DNA barcoding, but there is a lack of study of the combined sequences used as DNA barcodes in the two tree species. In this study, 10 single sequences and 4 combined sequences were selected for analysis, and the identification effect of each sequence was evaluated by the distance-based method, BLAST-based search, character-based method, and tree-based method.

**Results:**

Among the single sequences and the combined sequences, the interspecies distance of *trnH-psbA* and ITS2 + *trnH-psbA* was greater than the intraspecies distance, and there was no overlap in their frequency distribution plots. The results of the Wilcoxon signed-rank test for the interspecies distance of each sequence showed that the interspecies differences of the single sequences except *trnL-trnF*, *trnH-psbA*, and *ycf3* were significantly smaller than those of the combined sequences. The results of BLAST analysis showed that *trnH-psbA* could accurately identify *D. odorifera* and *D. tonkinensis* at the species level. In the character-based method, single sequences of *trnL-trnF*, *trnH-psbA* with all the combined sequences can be used for the identification of *D. odorifera* and *D. tonkinensis*. In addition, the neighbor-joining (NJ) trees constructed based on *trnH-psbA* and ITS2 + *trnH-psbA* were able to cluster *D. odorifera* and *D. tonkinensis* on two clades.

**Conclusions:**

The results showed that the character-based method with the BLOG algorithm was the most effective among all the evaluation methods, and the combined sequences can improve the ability to identify tree species compared with single sequences. Finally, the *trnH-psbA* and ITS2 + *trnH-psbA* were proposed as DNA barcodes to identify *D. odorifera* and *D. tonkinensis*.

**Supplementary Information:**

The online version contains supplementary material available at 10.1186/s12870-023-04513-3.

## Introduction

*Dalbergia odorifera* T. Chen is a semi-deciduous tree of the Leguminosae [1], which is endemic to Hainan Province, China, and has been introduced to Guangxi, Guangdong, and Yunnan [2]. Its heartwood has a beautiful color, excellent quality, and a unique aroma, which is often used in the production of valuable furniture [3]. In addition, *D. odorifera* also has various medicinal values, and modern pharmacological studies have shown that its ingredients have anti-inflammatory [4, 5], antioxidant [6, 7], blood-activating [8], anti-tumor [9, 10], anti-bacterial [11] and cardiovascular disease treatment effects [12, 13], etc. Due to its unique economic and medicinal value, the wild resources of *D. odorifera* have been severely damaged in recent years, and it was listed on the Red List of Threatened Species by the International Union for Conservation of Nature (IUCN) in 1998 [14].

In recent years, researchers have found that *Dalbergia tonkinensis* Prain from Vietnam is very similar to *D. odorifera* in terms of wood color, density, and even structural characteristics, but the two types of wood are widely disparate in price in the market [15, 16]. And unlike *D. odorifera*, the heartwood extract of *D. tonkinensis* has been shown to have anti-bacterial [17], anti-diabetic [18] and anti-hypertensive [19] properties. Due to the disparity in their economic and medicinal values, finding methods that can effectively identify them becomes especially important. It has been shown that they are difficult to distinguish by traditional methods such as morphological characteristics of the trees and structural characteristics of the wood [20, 21], and the chemical analysis results also show no significant differentiation in their chemical composition [22]. Therefore, it is difficult to distinguish *D. odorifera* from *D. tonkinensis* by the three methods mentioned above, and all of these methods require the extensive expertise of the evaluators.

DNA barcoding technology has shown powerful advantages in species identification, which can identify different species quickly and accurately, effectively solving the problem of relying on traditional methods that require a high level of evaluators. Currently, chloroplast genomic regions (such as *rbcL*, *matK*, *trnH-psbA*), as well as the internal transcribed spacer region (ITS) of nuclear ribosomal DNA, have become candidates for plant DNA barcoding [23, 24]. Some researchers used the sequences of *Dalbergia* spp. downloaded from the National Center of Biotechnology Information (NCBI) database for analysis and selected the ITS + *matK* + *rbcL* region as a DNA barcode to identify the species of this genus [25]; In addition, researchers have proposed different single sequences or combined sequences as DNA barcodes for the identification of *Dalbergia* species from different regions, including ITS2 + *trnH-psbA* [26], *matK* and *matK* + *rbcL* [27], ITS [28]. Highly variable regions in the chloroplast genome have also been shown to be useful for the identification of the trees of this genus [29–31]. In the DNA barcoding studies of *D. odorifera* and *D. tonkinensis*, some researchers suggested the *trnH-psbA* region as the DNA barcode to distinguish them [15], but other researchers have argued that the variable loci in this region are located in the palindromic region, which is not conserved among individuals making it unusable as a DNA barcode, thus they recommended the eight regions selected from the chloroplast genome as DNA barcodes for the two tree species [32]. The above analysis shows that the results of DNA barcoding studies on *Dalbergia* species, especially *D. odorifera* and *D. tonkinensis*, are still not consistent, and the combined sequences as DNA barcodes have not attracted much attention in the studies of these two species.

In this study, 10 single sequences (ITS2, *rpoB*, *rpoC1*, *trnH-psbA*, *trnL-trnF*, *matK*, *ycf3*, *trnL intron*, *trnS-psbC*, *rbcL*) and 4 combined sequences were analyzed by various analysis methods. The purpose was to compare different analysis methods and to investigate whether the combination of sequences could improve the identification abilities of *D. odorifera* and *D. tonkinensis*, and finally to select the optimal DNA barcode region for the identification of the two species.

## Materials and methods

### Plant materials

A total of 38 plant leaf materials were collected from China and Vietnam. Among them, all 16 *D. odorifera* materials were collected from southern China, and 16 of the *D. tonkinensis* materials were collected from Vietnam and 6 from China. The sampling information is shown in Table 1.

Among the leaf materials of *D. odorifera*, H1-5 were obtained from the Experimental Center of Tropical Forestry Chinese Academy of Forestry in Pingxiang, Guangxi, H6-10 from Nanning Arboretum in Nanning, Guangxi, H11-13 from Fengmu Forestry Farm in Fengmu Town, Tunchang County, Hainan, and H14-16 from Haikou People’s Park in Haikou, Hainan. Among the materials of *D. tonkinensis*, Y1 was sourced from the Qinzhou Port Wood Storage Plant in Qinzhou, Guangxi, Y2-7 from the Hanoi Botanical Garden in Hanoi, Vietnam, Y8-12 from street trees in Nanshan Town, Pingxiang, Guangxi, Y13-18 from street trees in Tongji Village, Bac Ninh, Vietnam, and Y19-22 from the experimental forestry farm of Vietnam Forestry University in Quang Ninh, Vietnam. All plant materials have been formally identified by Professor Xu Feng (Guangxi University Forest Products Quality Testing Center). The voucher specimens are preserved in the Wood Herbarium of the College of Forestry, Guangxi University, and the specimen information is shown in Table S1. All the collected materials require no specific permission or license.

**Table 1:** Information of the plant samples

Species Name	Sample No.	Collection locations	Sample Types
*Dalbergia odorifera*	H1-5	Pingxiang, Guangxi, China	Cultivated
*D. odorifera*	H6-10	Nanning, Guangxi, China	Cultivated
*D. odorifera*	H11-13	Tunchang, Hainan, China	Wild
*D. odorifera*	H14-16	Haikou, Hainan, China	Cultivated
*Dalbergia tonkinensis*	Y1	Qinzhou, Guangxi, China	Cultivated
*D. tonkinensis*	Y2-7	Ha Noi, Viet Nam	Cultivated
*D. tonkinensis*	Y8-12	Pingxiang, Guangxi, China	Cultivated
*D. tonkinensis*	Y13-18	Tu Son, Bac Ninh, Viet Nam	Cultivated
*D. tonkinensis*	Y19-22	Dong Trieu, Quang Ninh, Viet Nam	Wild

### DNA extraction, amplification, and sequencing

DNA was extracted using the DNeasy Plant Mini Kit (Qiagen), and the extracted DNA was stored at -20℃. Ten DNA sequences (ITS2, *rpoB*, *rpoC1*, *trnH-psbA*, *trnL-trnF*, *matK*, *ycf3*, *trnL intron*, *trnS-psbC*, *rbcL*) were selected as candidate barcodes for primer design, and the sequences and primer information are shown in Table S2. PCR amplification was performed in a 25 µL system, and the amplified products were first detected by 1% agarose gel electrophoresis, then purified by Biospin PCR product purification kit, and the purified products were sequenced by Beijing Liuhe Huada Gene Technology Co., Ltd (China). The GeneBank accession numbers of each sequence are shown in Table S3.

### Data analysis

Multiple comparisons of sequences were performed using MEGA 11 software, and the basic information of each sequence was counted after manual adjustment, after which the sequences with differences were selected for further analysis. In addition, four combinations of *trnL-trnF* + ITS2, *trnL-trnF* + *trnH-psbA*, ITS2 + *trnH-psbA*, and *trnL-trnF* + ITS2 + *trnH-psbA*, which were selected based on the discrimination ability of single sequences in this study, were used as candidate DNA barcodes together for subsequent identification analysis.

The ideal DNA barcode should have low intraspecific and high interspecific variations [33]. The Kimura two-parameter (K2P) model in MEGA 11 software was used to calculate interspecific and intraspecific genetic distances. Interspecific genetic variation was assessed by the average distance, and intraspecific variation was assessed by three parameters: average intraspecific distance, theta (θ), and average coalescence depth. Subsequently, the barcoding gap was assessed based on the genetic distance distribution plots of inter- and intraspecies distances. The sequences that can be used as DNA barcodes should exhibit independent, non-overlapping distributions of genetic variation in intra- and interspecific samples [34]. At last, the Wilcoxon signed-rank test was performed by IBM SPSS Statistics 27 to confirm whether there were significant interspecies differences among the different sequences.

The identification ability of each sequence was further evaluated by the BLAST-based method, character-based method, and tree-based method. All sequences were aligned in the NCBI database by BLAST sequence similarity search, and the best match was selected as the identification results with E value < cut-off value (E value < 10^− 6^) according to Ross et al. [35]. Barcoding with LOGic is an analysis method based on sequence characteristics, and in this study, the BLOG 2.0 software was used to evaluate the discrimination rate of different sequences and to obtain logical rules for the discrimination of each sequence. The MEGA 11 software was used to construct the neighbor-joining (NJ) trees, the bootstrap supporting option was set to 1000 random addition replicates to determine statistical support for the clades. When all individuals of the same species can be clustered in a single clade, the species is considered to be successfully identified.

## Results

### Sequence features

Multiple alignment was performed for all sequences and the information of each sequence is shown in Table 2. The length of single sequences ranged from 256 bp (*trnH-psbA*) to 866 bp (*matK*), and there were no variable sites in *rpoB*, *rpoC1*, and *rbcL*, indicating that these three sequences did not apply to the identification of *D. odorifera* and *D. tonkinensis*. Among the other sequences, *trnL-trnF* had the highest proportion of informative sites (66/409 bp), followed by *trnH-psbA* (7/256 bp), *ycf3* (18/690 bp), ITS2 (11/558), *trnS-psbC* (4/347), and *trnL* intron (2/519), and the lowest is *matK* (1/866 bp). The combined sequences ranged from 665 bp (*trnL-trnF* + *trnH-psbA*) to 1223 bp (*trnL-trnF* + ITS2 + *trnH-psbA*) in length, and the order of the proportion of informative sites for each sequence was *trnL-trnF* + *trnH-psbA* (70/665), *trnL-trnF* + ITS2 (69/967), *trnL-trnF* + ITS2 + *trnH-psbA* (73/1223), and ITS2 + *trnH-psbA* (18/814).

**Table 2:** Statistics of sequence characteristics

Sequences	No. of sequences analyzed	Aligned sequence length (bp)	No. of conserved sites	No. of variable sites	No. of informative sites	G + C ratio (%)
ITS2	37	558	547	11	11	63.73
*rpoB*	36	510	510	0	0	41.35
*rpoC1*	36	494	494	0	0	40.08
*trnH-psbA*	33	256	249	7	7	27.56
*trnL-trnF*	14	409	314	95	66	35.43
*matK*	16	866	854	8	1	36.62
*ycf3*	15	690	647	41	18	28.79
*trnL* intron	30	519	517	2	2	34.09
*trnS-psbC*	29	347	338	9	4	39.21
*rbcL*	29	676	676	0	0	42.75
*trnL-trnF* + ITS2	14	967	864	103	69	51.86
*trnL-trnF* + *trnH-psbA*	13	665	565	100	70	32.31
ITS2 + *trnH-psbA*	30	814	793	21	18	52.34
*trnL-trnF* + ITS2 + *trnH-psbA*	12	1223	1115	108	73	46.72

## Distance analysis and barcoding gap assessment

The statistics of the K2P distances of single sequences and combined sequences are shown in Table 3. The interspecific distances ranged from 0.0005 to 0.0828 and intraspecific distances ranged from 0.0000 to 0.0652 in the single sequences, and the inter- and intraspecific distances can be used to assess the genetic variation of the sequences. Among them, *trnL-trnF* had the highest interspecific and intraspecific genetic variation, *trnL* intron had the lowest interspecific genetic variation, and *trnH-psbA* had the lowest intraspecific genetic variation. In addition, the average interspecific distances of all sequences except *ycf3* and *trnL* intron were higher than the average intraspecific distances. The interspecies distances of the combined sequences ranged from 0.0137 to 0.0626 and intraspecies distances from 0.0036 to 0.0387. Among them, *trnL-trnF* + *trnH-psbA* had the highest inter- and intraspecific genetic variation, and ITS2 + *trnH-psbA* had the lowest inter- and intraspecific variation.

**Fig. 1:**
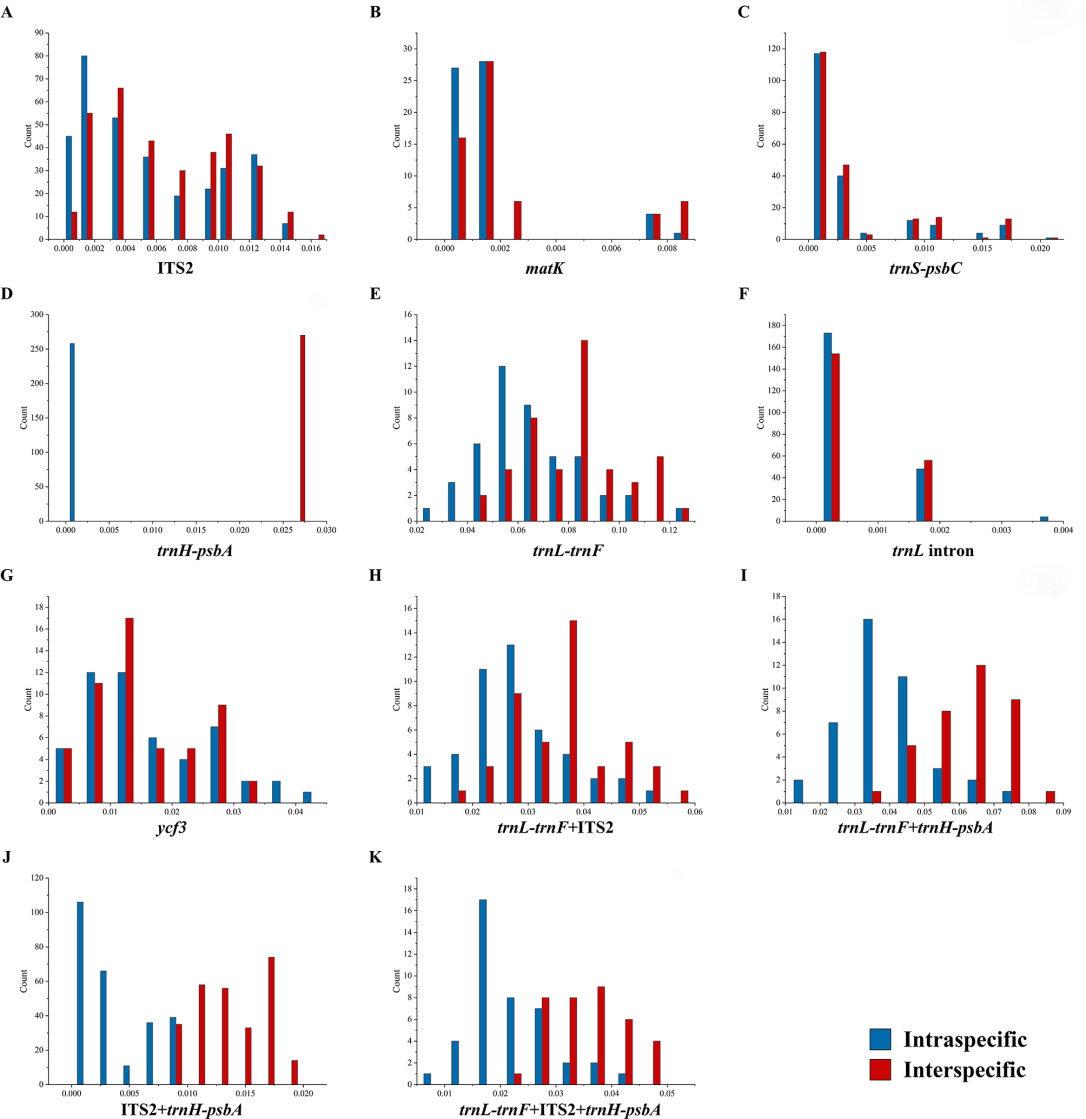
Distribution of interspecific and intraspecific distances of sequences (**A**: ITS2; **B**: *matK*; **C**: *trnS-psbC*; **D**: *trnH-psbA*; **E**: *trnL-trnF*; **F**: *trnL* intron; **G**: *ycf3*; **H**: *trnL-trnF* + ITS2; **I**: *trnL-trnF* + *trnH-psbA*; **J**: ITS2 + *trnH-psbA*; **K**: *trnL-trnF* + ITS2 + *trnH-psbA*)

The frequency distributions of inter- and intraspecific distances for single and combined sequences are shown in Fig. 1. The results indicated that the interspecific variation of *trnH-psbA* was greater than the intraspecific variation, forming a clear gap (Fig. 1-D), while the distribution of inter- and intraspecific distances of the rest of the single sequences showed some overlaps. Among the combined sequences, the distribution of inter- and intraspecific distances did not overlap in ITS2 + *trnH-psbA* (Fig. 1-J) and overlapped in the rest of the sequences.

**Table 3:** Interspecific and intraspecific distance analysis of sequences based on the Kimura two-parameter model

Sequences	Interspecific and intraspecific distance analysis			
	Average interspecific distances	Average intraspecific distance	Theta(θ)	Average coalescence depth
ITS2	0.0067 ± 0.0002	0.0054 ± 0.0002	0.0046 ± 0.0027	0.0109 ± 0.0037
*matK*	0.0021 ± 0.0003	0.0012 ± 0.0003	0.0017 ± 0.0011	0.0047 ± 0.0035
*trnS-psbC*	0.0033 ± 0.0004	0.0030 ± 0.0004	0.0028 ± 0.0024	0.0118 ± 0.0089
*trnH-psbA*	0.0279 ± 0.0000	0.0000 ± 0.0000	0.0000 ± 0.0000	0.0000 ± 0.0000
*trnL-trnF*	0.0828 ± 0.0029	0.0652 ± 0.0030	0.0732 ± 0.0141	0.1084 ± 0.0194
*trnL* intron	0.0005 ± 0.0001	0.0005 ± 0.0001	0.0005 ± 0.0005	0.0020 ± 0.0020
*ycf3*	0.0154 ± 0.0012	0.0161 ± 0.0014	0.0137 ± 0.0060	0.0151 ± 0.0214
*trnL-trnF* + ITS2	0.0362 ± 0.0013	0.0283 ± 0.0013	0.0319 ± 0.0064	0.0450 ± 0.0050
*trnL-trnF* + *trnH-psbA*	0.0626 ± 0.0020	0.0387 ± 0.0019	0.0464 ± 0.0107	0.0650 ± 0.0150
ITS2 + *trnH-psbA*	0.0137 ± 0.0002	0.0036 ± 0.0002	0.0033 ± 0.0019	0.0074 ± 0.0025
*trnL-trnF* + ITS2 + *trnH-psbA*	0.0356 ± 0.0012	0.0219 ± 0.0011	0.0265 ± 0.0065	0.0365 ± 0.0064

### Wilcoxon signed-rank test analysis

The interspecies distances of single and combined sequences were analyzed with the Wilcoxon signed-rank test and the results are shown in Tables 4 and 5. The interspecific distances of all sequences in the single sequences were significantly different, in the order of *trnL-trnF* > *trnH-psbA* > *ycf3* > ITS2 > *trnS-psbC* > *matK* > *trnL* intron. The test results for the combined sequences were *trnL-trnF* + *trnH-psbA* > *trnL-trnF* + ITS2, *trnL-trnF* + ITS2 + *trnH-psbA* > ITS2 + *trnH-psbA*. And trnL-trnF + ITS2 and *trnL-trnF* + ITS2 + *trnH-psbA* could not be compared as there was no significant difference (p = 0.9000) between them. In addition, the interspecies distances of single and combined sequences were also tested in this study (Table S4). The results showed that among the single sequences, the interspecies differences of *trnL-trnF* were higher than all the combined sequences, *trnH-psbA* > ITS2 + *trnH-psbA*, and no significant differences existed between *ycf3* and ITS2 + *trnH-psbA* (p = 0.295) thus cannot be compared. Except for these, the interspecies differences of all the single sequences were significantly lower than the combined sequences.

**Table 4:** Wilcoxon signed-rank test for the interspecies distances of the single sequences

W^+^	W^−^	Relative Ranks		n	p	results
ITS2	*matK*	W^+^=1631.00	W^−^=139.00	60	0.000	ITS2 > *matK*
ITS2	*trnS-psbC*	W^+^=16282.00	W^−^=4833.00	210	0.000	ITS2 > *trnS-psbC*
ITS2	*trnH-psbA*	W^+^=0.00	W^−^=36585.00	270	0.000	ITS2 < *trnH-psbA*
ITS2	*trnL-trnF*	W^+^=0.00	W^−^=1035.00	45	0.000	ITS2 < *trnL-trnF*
ITS2	*trnL* intron	W^+^=20540.00	W^−^=166.00	210	0.000	ITS2 > *trnL* intron
ITS2	*ycf3*	W^+^=91.00	W^−^=1394.00	54	0.000	ITS2 < *ycf3*
*matK*	*trnS-psbC*	W^+^=547.00	W^−^=831.00	60	0.000	*matK* < *trnS-psbC*
*matK*	*trnH-psbA*	W^+^=0.00	W^−^=1830.00	60	0.000	*matK* < *trnH-psbA*
*matK*	*trnL-trnF*	W^+^=0.00	W^−^=1035.00	45	0.000	*matK* < *trnL-trnF*
*matK*	*trnL* intron	W^+^=993.00	W^−^=183.00	60	0.000	*matK* > *trnL* intron
*matK*	*ycf3*	W^+^=0.00	W^−^=1485.00	54	0.000	*matK* < *ycf3*
*trnS-psbC*	*trnH-psbA*	W^+^=0.00	W^−^=22155.00	210	0.000	*trnS-psbC* < *trnH-psbA*
*trnS-psbC*	*trnL-trnF*	W^+^=0.00	W^−^=1035.00	45	0.000	*trnS-psbC* < *trnL-trnF*
*trnS-psbC*	*trnL* intron	W^+^=6735.00	W^−^=450.00	210	0.000	*trnS-psbC* > *trnL* intron
*trnS-psbC*	*ycf3*	W^+^=8.00	W^−^=1477.00	54	0.000	*trnS-psbC* < *ycf3*
*trnH-psbA*	*trnL-trnF*	W^+^=0.00	W^−^=1035.00	45	0.000	*trnH-psbA* < *trnL-trnF*
*trnH-psbA*	*trnL* intron	W^+^=22155.00	W^−^=0.00	210	0.000	*trnH-psbA* > *trnL* intron
*trnH-psbA*	*ycf3*	W^+^=1427.00	W^−^=48.00	54	0.000	*trnH-psbA* > *ycf3*
*trnL-trnF*	*trnL* intron	W^+^=1035.00	W^−^=0.00	45	0.000	*trnL-trnF* > *trnL* intron
*trnL-trnF*	*ycf3*	W^+^=1035.00	W^−^=0.00	45	0.000	*trnL-trnF* > *ycf3*
*trnL* intron	*ycf3*	W^+^=1.00	W^−^=1484.00	54	0.000	*trnL* intron < *ycf3*

**Table 5:** Wilcoxon signed-rank test for the interspecies distances of the combined sequences

W^+^	W^−^	Relative Ranks			n	p	results
*trnL-trnF* + ITS2	*trnL-trnF* + *trnH-psbA*	W^+^= 1.00	W^−^=665.00		36	0.000	*trnL-trnF* + ITS2 < *trnL-trnF* + *trnH-psbA*
*trnL-trnF* + ITS2	ITS2 + *trnH-psbA*	W^+^=1035.00	W^−^=0.00		45	0.000	*trnL-trnF* + ITS2 > ITS2 + *trnH-psbA*
*trnL-trnF* + ITS2	*trnL-trnF* + ITS2 + *trnH-psbA*	W^+^=341.00	W^−^=325.00		36	0.900	*trnL-trnF* + ITS2 > *trnL-trnF* + ITS2 + *trnH-psbA*
*trnL-trnF* + *trnH-psbA*	ITS2 + *trnH-psbA*	W^+^=666.00	W^−^=0.00		36	0.000	*trnL-trnF* + *trnH-psbA* > ITS2 + *trnH-psbA*
*trnL-trnF* + *trnH-psbA*	*trnL-trnF* + ITS2 + *trnH-psbA*	W^+^=666.00	W^−^=0.00		36	0.000	*trnL-trnF* + *trnH-psbA* > *trnL-trnF* + ITS2 + *trnH-psbA*
ITS2 + *trnH-psbA*	*trnL-trnF* + ITS2 + *trnH-psbA*	W^+^=0.00	W^−^=666.00		36	0.000	ITS2 + *trnH-psbA* < *trnL-trnF* + ITS2 + *trnH-psbA*

### BLAST search-based analysis

The identification rates of different sequences were compared based on the BLAST search. For single sequences, the identification rates of ITS2, *matK*, *trnH-psbA*, and *trnL* intron were in the range of 99-100%. Besides, among the *trnS-psbC* sequences, 3 sequences were identified at less than 99%, and the other 26 sequences were identified at 99-100%; among the sequences of *trnL-trnF*, 5 sequences were identified at 90-95%, 2 sequences were identified at less than 90%, and the rest 7 sequences were identified at 100%; and among the sequences of *ycf3*, 9 sequences were identified at less than 99% and 6 sequences were identified at 99-100%. The identification rates were 99-100% for all of the combined sequences.

The analysis of the sequence identification results is shown in Table 6. The identification results at the genus level showed that the identification success rate of *matK* was 0%, the identification success rate of *trnL-trnF* was 42.9%, and the identification error rate was 57.1%, all the remaining sequences were 100% in the identification success rate. At the species level, the identification success rate of *trnH-psbA* was 100%, which means that this sequence can achieve accurate identification at the species level. Among the other single sequences, the identification success rate of ITS2 was 67.6%, the error rate was 24.3% and the ambiguous identification rate was 8.1%; the identification success rate of *ycf3* was 53.3%, the error rate was 26.7% and the ambiguous identification rate was 20%, while the identification success rates of *matK*, *trnS-psbC*, *trnL-trnF*, and *trnL* intron were all 0%. Among the combined sequences, the highest identification success rate was *trnL-trnF* + ITS2 + *trnH-psbA* with 76.9%, and the others were followed by *trnL-trnF* + ITS2 (71.4%), ITS2 + *trnH-psbA* (69.7%), and *trnL-trnF* + *trnH-psbA* (30.8%). The above results show that among all sequences, *trnH-psbA* can accurately distinguish *D. odorifera* and *D. tonkinensis* by the BLAST-based method.

**Table 6:** BLAST analysis of sequences at the genus and species level

Sequences	Identification success rate (%)		Identification error rate (%)		Ambiguous identification rate (%)	
	Species level	Genus level	Species level	Genus level	Species level	Genus level
ITS2	67.6% (25/37)	100% (37/37)	24.3% (9/37)	0 (0/37)	8.1% (3/37)	0 (0/37)
*matK*	0 (0/16)	0 (0/16)	100% (16/16)	100% (16/16)	0 (0/16)	0 (0/16)
*trnS-psbC*	0 (0/29)	100% (29/29)	17.2% (5/29)	0 (0/29)	82.8% (24/29)	0 (0/29)
*trnH-psbA*	100% (33/33)	100% (33/33)	0 (0/33)	0 (0/33)	0 (0/33)	0 (0/33)
*trnL-trnF*	0 (0/14)	42.9% (6/14)	100% (14/14)	57.1% (8/14)	0 (0/14)	0 (0/14)
*trnL* intron	0 (0/30)	100% (30/30)	0 (0/30)	0 (0/30)	100% (30/30)	0 (0/30)
*ycf3*	53.3% (8/15)	100% (15/15)	26.7% (4/15)	0 (0/15)	20% (3/15)	0 (0/15)
*trnL-trnF* + ITS2	71.4% (10/14)	100% (14/14)	21.4% (3/14)	0 (0/14)	7.1% (1/14)	0 (0/14)
*trnL-trnF* + *trnH-psbA*	30.8% (4/13)	100% (13/13)	0 (0/13)	0 (0/13)	69.2% (9/13)	0 (0/13)
ITS2 + *trnH-psbA*	69.7% (23/33)	100% (33/33)	21.2% (7/33)	0 (0/33)	9.1% (3/33)	0 (0/33)
*trnL-trnF* + ITS2 + *trnH-psbA*	76.9% (10/13)	100% (13/13)	15.4% (2/13)	0 (0/13)	7.7% (1/13)	0 (0/13)

Notes: Identification success rate (%): percentage of the sequences with the highest similarity of the target species to the same species after the BLAST search; Identification error rate (%): percentage of the sequences with the highest similarity to different species but lower similarity to the same species after the BLAST search; Ambiguous identification rate (%): percentage of the sequences in which the target species had the highest similarity to both the same species and different species after searching by BLAST.

### Character-based analysis of sequences

The identification rate and logic formulae of each sequence based on the BLOG algorithm are shown in Table 7. The correct classification rate of *trnL-trnF*, *trnH-psbA*, and all the combined sequences for *D. odorifera* and *D. tonkinensis* was 100%. Based on *trnL-trnF*, if locus 243 was A and locus 375 was T, then the sequence belonged to *D. odorifera*, and if locus 322 was C and locus 373 was C, then the sequence belonged to *D. tonkinensis*. Based on *trnH-psbA*, if locus 196 was A, it belonged to *D. odorifera*, and if the locus was C, it belonged to *D. tonkinensis*. The logic formula for the identification of *trnL-trnF* + ITS2 in the combined sequence was the summary of these two single sequences, when the sequence with T at locus 375 and A at locus 412, it belonged to *D. odorifera*, and with C at locus 322 and C at locus 373, it belonged to *D. tonkinensis*. The logic formulae for the identification of the other three combined sequences were consistent with *trnH-psbA*. This result shows that *trnL-trnF*, *trnH-psbA*, and all the combined sequences can accurately identify *D. odorifera* and *D. tonkinensis* by the character-based method.

**Table 7:** Character-based approach for species identification

Sequences	cc	wc	nc	Formula	
				*Dalbergia odorifera*	*Dalbergia tonkinensis*
ITS2	88.89	11.11	0.00	3 = A 8 = C 500 = C	19 = G 59 = C 63 = G
*matK*	50.00	50.00	0.00	866 = C	866 = T
*trnS-psbC*	0.00	0.00	100.00	338 = C	338 = C
*trnH-psbA*	100.00	0.00	0.00	196 = A	196 = C
*trnL-trnF*	100.00	0.00	0.00	243 = A 375 = T	322 = C 373 = C
*trnL* intron	0.00	0.00	100.00		
*ycf3*	50.00	50.00	0.00	672 = G or 482 = G 679 = C	672 = T 679 = T or 482 = A 672 = T
*trnL-trnF* + ITS2	100.00	0.00	0.00	375 = T 412 = A	322 = C 373 = C
*trnL-trnF* + *trnH-psbA*	100.00	0.00	0.00	605 = A	605 = C
ITS2 + *trnH-psbA*	100.00	0.00	0.00	754 = A	754 = C
*trnL-trnF* + ITS2 + *trnH-psbA*	100.00	0.00	0.00	1163 = A	1163 = C

Notes: cc: correctly classified, wc: wrongly classified, nc: not classified

### Tree-based analysis

**Fig. 2:**
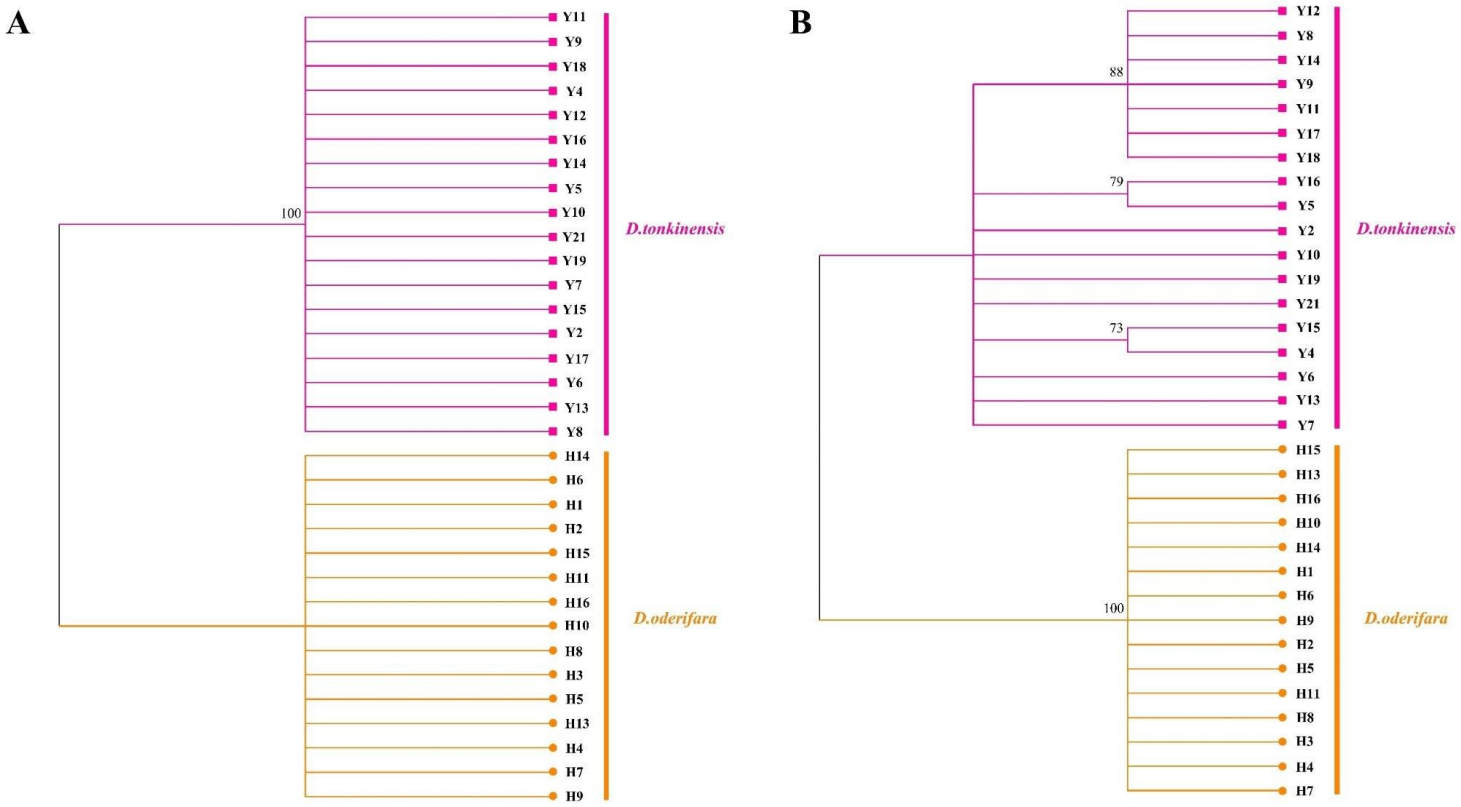
NJ trees constructed based on *trnH-psbA* and ITS2 + *trnH-psbA* (**A**: *trnH-psbA*; **B**: ITS2 + *trnH-psbA*)

The NJ trees were constructed separately with single and combined sequences. The results showed that the NJ tree constructed based on *trnH-psbA* was able to separate *D. odorifera* and *D. tonkinensis* (Fig. 2-A). The samples of these two tree species were clustered on two clades with a support value of 100%. Among the other sequences, the construction of NJ trees based on *trnL-trnF* with the greatest interspecific variation revealed that Y1, Y4, and Y5 in *D. tonkinensis* were able to cluster on the same clade with 88% support, but Y6 and Y7 clustered in a clade with the samples of *D. odorifera* (Fig. S1). It showed that the NJ tree constructed by *trnL-trnF* could not identify *D. odorifera* and *D. tonkinensis*.

Among the combined sequences, ITS2 + *trnH-psbA* was able to be used for the identification of *D. odorifera* and *D. tonkinensis* (Fig. 2-B). In the NJ tree constructed based on this sequence, the samples of *D. odorifera* and *D. tonkinensis* clustered in two clades, and the clade clustered by the samples of *D. odorifera* had 100% support. The NJ tree constructed based on *trnL-trnF* + ITS2 showed similar results to that of *trnL-trnF*, with Y1, Y4, and Y5 in *D. tonkinensis* able to cluster in the same clade with 83% support, while Y6 and Y7 clustered with the samples of *D. odorifera* (Fig. S2). In the NJ trees constructed based on *trnL-trnF* + *trnH-psbA* and *trnL-trnF* + *trnH-psbA* + ITS2, Y6 and Y7 in *D. tonkinensis* were also clustered in the same clade as the samples of *D. odorifera*, but all the samples of *D. odorifera* were able to be clustered together with more than 90% support (Fig. S3, S4). Therefore, none of these three combined sequences could be used for the identification of *D. odorifera* and *D. tonkinensis*.

The above results showed that *trnH-psbA* in single sequences and ITS2 + *trnH-psbA* in combined sequences could identify *D. odorifera* and *D. tonkinensis* by the method of constructing NJ trees.

## Discussions

### Comparison of DNA barcode analysis methods

In the current study, the main DNA barcode analysis methods include distance-based method, BLAST-based method, character-based method, and tree-based method. In addition, there are other methods that use DNA barcoding software, such as best match (BM) and best close match (BCM) analysis in TAXONDNA, to evaluate the identification effect [36]. All of the above analysis methods have been applied in the study of *Dalbergia* species, but the character-based method has not been reported in the studies of *D. odorifera* and *D. tonkinensis*. However, these analysis methods have not been evaluated either in *Dalbergia* species or in studies of these two tree species, making it difficult to find the most convenient and effective identification method for practical application. Therefore, in this study, the character-based method via BLOG was added to the methods of the distance-based method, BLAST-based method, and tree-based method, and the role of these four analysis methods in the selection of DNA barcodes in *D. odorifera* and *D. tonkinensis* was also evaluated.

The distance-based method is commonly used to assess interspecific and intraspecific variation in sequences and use their distribution to determine the barcoding gap. And some researchers will compare the interspecies variation of different sequences by the Wilcoxon signed-rank test for further analysis [27]. Combining the genetic distance distribution of each sequence with the results of the Wilcoxon signed-rank test, *trnH-psbA* and ITS2 + *trnH-psbA* were recommended as DNA barcodes of *D. odorifera* and *D. tonkinensis*. However, this method could not evaluate the identification effect of barcodes.

BLAST is an analysis method based on the similarity comparison of sequences. Ross et al. used four different algorithms to evaluate the effect of species identification based on the BLAST method, with the BLAST1 algorithm having the highest species identification rate [35]. This study also used the algorithm of BLAST1 to evaluate the identification effect of DNA barcodes. Among all sequences, all 9 sequences except *matK* and *trnL-trnF* could be accurately compared to the species of *Dalbergia*, but only *trnH-psbA* could identify *D. odorifera* and *D. tonkinensis*. It shows that the BLAST-based method has a high identification rate at the genus level, but it is difficult to achieve accurate identification at the species level, and this conclusion is consistent with the results of previous studies [36, 37].

The character-based method is used to identify species by the presence or absence of discrete nucleotide substitutions (character states) in a sequence [38]. This analysis method can be implemented by the Characteristic Attributes Organization System (CAOS) algorithm [38] or the BLOG algorithm [39]. This method was used for DNA barcode selection in the study of *Dalbergia* species [40]. In this study, the DNA barcodes of *D. odorifera* and *D. tonkinensis* were analyzed by the BLOG algorithm, in which the accurate identification rate of ITS2 was over 80%, while *trnL-trnF*, *trnH-psbA*, and four combined sequences were able to accurately identify these two species.

Methods based on the reconstruction of phylogenetic relationships usually use methods such as NJ, MP, or UPGMA to construct phylogenetic trees, and the clustering of each species in the tree is used as the identification basis for the evaluation of DNA barcodes. However, some researchers consider that the evolutionary relationships of species based on phylogenetic trees constructed with DNA barcodes are unreliable because the sequences used as DNA barcodes are usually short [41]. In addition, some researchers have argued that using this method in the classification of paraphyletic groups or incomplete lineages may produce incorrect or ambiguous identification results [42]. The results of this study showed that *trnH-psbA*, ITS2 + *trnH-psbA* were able to cluster samples of *D. odorifera* and *D. tonkinensis* in two clades by constructing NJ trees, indicating that this method can be used for DNA barcode evaluation in the identification of these two tree species.

In summary, the distance-based method cannot be used to directly assess the identification effect of DNA barcodes, but it can be used as one of the bases for selection. The BLAST-based method is suitable for identification at the genus level but cannot be used for identification at the species level. Both character-based and tree-based methods can evaluate the identification effect of DNA barcodes. In this study, the sequences showed the best identification effect using the character-based method with the BLOG algorithm.

### Comparison of the discriminatory ability of single sequences and combined sequences

Based on current studies of genome structure, nucleotide substitution rates, and variation in rate distribution, it is difficult to find a DNA barcode that is suitable for all plants [43]. Since Kress proposed the idea of sequence combination, a growing number of studies have demonstrated that combined sequences have higher discriminatory power in species than single sequences [23]. In the study of *Dalbergia* species, Yu et al. analyzed 8 barcode sequences and their combinations of 9 species of this genus and found that the combined sequences had higher identification power than any single sequence [26]. Similarly, other researchers also proposed the combined sequence as the DNA barcodes for this genus [25, 40, 44]. However, the combination of sequences as DNA barcodes did not attract much attention in the studies of *D. odorifera* and *D. tonkinensis*, and the researchers tested only the identification abilities of single sequences in the studies of the two species [15]. In this study, in addition to evaluating the single sequences, three sequences, *trnL-trnF*, ITS2, and *trnH-psbA*, were selected for combination based on the discrimination ability of the single sequences, and the four combined sequences were evaluated for their discrimination abilities in *D. odorifera* and *D. tonkinensis*.

Seven single sequences and four combined sequences of *D. odorifera* and *D. tonkinensis* were analyzed in this study. The results of the analysis based on the distance method showed that the mean interspecies distances of the combined sequences were higher than those of the other single sequences except for *trnL-trnF* and *trnH-psbA*, and the results of the Wilcoxon signed-rank test also supported this conclusion. However, the results of the genetic distance distribution showed that only the inter- and intraspecific distances of ITS2 + *trnH-psbA* in the combined sequences did not show overlapping regions. The results of BLAST-based analysis showed that the combined sequences could improve the identification success rate to a certain extent compared with single sequences, but the method could not be used for identification at the species level. The analysis of the character-based method showed that only two of the single sequences were able to accurately identify *D. odorifera* and *D. tonkinensis*, while all combinations of sequences were able to achieve this goal. According to the logical formulas given by the BLOG algorithm, the logical formulas of the three combined sequences were consistent with *trnH-psbA* in the single sequences, indicating that the sequence *trnH-psbA* may still play a major role in these combined sequences. But this still indicates a significant improvement in the identification ability of the combined sequences compared to the single sequences. However, this conclusion was not supported by the results of the tree-based analysis. In addition to the *trnH-psbA* sequence in the single sequence, only the NJ tree constructed based on ITS2 + *trnH-psbA* was observed to cluster the samples of *D. odorifera* and *D. tonkinensis* on two clades among all combined sequences, while the others could not accurately identify the two species.

Therefore, the results of this study indicate that the combined sequences have higher identification abilities for *D. odorifera* and *D. tonkinensis* compared to single sequences. However, not all of the combined sequences were able to accurately identify the two species, which was also related to the selection of the analysis methods.

### **Selection of DNA barcodes for *Dalbergia odorifera *and *D. tonkinensis***

In previous studies, there was some controversy among different researchers about whether *trnH-psbA* could be used as the DNA barcode of *D. odorifera* and *D. tonkinensis*. Although Qin et al. suggested that the variable loci in *trnH-psbA* are located in the palindromic region which is not conserved in different individuals, they did not perform experiments to verify this inference [15, 32]. In this study, four analysis methods, including distance-based method, BLAST-based method, character-based method, and tree-based method, were used to evaluate whether the sequence *trnH-psbA* could be used as a DNA barcode for *D. odorifera* and *D. tonkinensis*. The chloroplast genome sequences such as *rpoB*, *trnL* intron, *trnL-trnF*, *ycf3*, and *trnS-psbC*, which have not been used in the study of *D. odorifera* and *D. tonkinensis* were added to this study, the aim was to select new DNA barcodes from these sequences that are suitable for distinguishing these two tree species.

In this study, based on the results of genetic distance distribution, the interspecific variation of *trnH-psbA* was greater than the intraspecific variation and formed a clear gap. The BLAST-based analysis showed that the sequence was able to accurately identify the two tree species at the species level. The results of the analysis based on sequence characteristics also showed that the accurate identification of these two species by this sequence was 100%. The samples of *D. odorifera* and *D. tonkinensis* were able to cluster in two different clades in the NJ tree constructed based on *trnH-psbA*. The experimental results obtained from multiple evaluation methods demonstrated the strong identification ability of *trnH-psbA*, providing powerful support for the conclusion that it can be used as a DNA barcode for these two species. Except for *trnH-psbA*, ITS2 + *trnH-psbA* was found to be able to distinguish *D. odorifera* and *D. tonkinensis* in all analyses other than those of the BLAST-based method in the study of combined sequences. Moreover, it was demonstrated in previous studies that this combined sequence can be used for the identification of *Dalbergia* species, which is consistent with the results of this study [26]. In summary, *trnH-psbA* and ITS2 + *trnH-psbA* are recommended as DNA barcodes for the identification of *D. odorifera* and *D. tonkinensis*.

In addition, in the studies of *Dalbergia* species, different researchers have proposed different sequences as DNA barcodes to distinguish the species of this genus, such as *matK* [27], ITS [28] in single sequences, and *matK* + *rbcL* [27], ITS + *matK* + *rbcL* [25] in combined sequences. In this study, the identification of some of the above sequences in *D. odorifera* and *D. tonkinensis* was verified. Among them, the sequencing results of *rbcL* showed that there were no variable sites in this sequence of *D. odorifera* and *D. tonkinensis*, and the lowest proportion of informative sites in *matK*, both of which and the related combined sequences could not be used as DNA barcodes for these two species, which was confirmed by the further analysis results of *matK*. The above results are consistent with the previous conclusion that these sequences cannot identify closely related tree species [32]. As for other candidate regions suitable for plants or wood identification, such as *ycf3* and *trnS-psbC*, the analysis results of this study proved that they could not be used for the identification of *D. odorifera* and *D. tonkinensis*.

## Conclusions

In this study, four methods were used to assess the identification abilities of 10 single sequences and 4 combined sequences for *D. odorifera and D. tonkinensis*. Among all methods, sequences showed the best identification effect using the character-based method with the BLOG algorithm. Moreover, compared with single sequences, the combined sequences can improve the identification abilities of tree species to a certain extent. In addition, the experimental results demonstrated that *trnH-psbA* in the single sequences were able to accurately distinguish *D. odorifera and D. tonkinensis* by the above four evaluation methods, while in the analysis of the combined sequences, ITS2 + *trnH-psbA* showed good identification ability. Both sequences were able to be used as DNA barcodes for these two species.

### Electronic supplementary material

Below is the link to the electronic supplementary material.

Supplementary Material 1

Supplementary Material 2

Supplementary Material 3

Supplementary Material 4

Supplementary Material 5

## Data Availability

The original contributions presented in the study are included in the article/supplementary material, and the GeneBank accession numbers of each sequence are shown in Table S3. Further inquiries can be directed to the corresponding authors.
