# Supplementary material for: The DNA barcode identification of Dalbergia odorifera T. Chen and Dalbergia tonkinensis Prain
Source: BMC Plant Biol. 2023 Nov 7;23:546. doi: 10.1186/s12870-023-04513-3 (PMC10629101; doi:10.1186/s12870-023-04513-3)
Supplement: Supplementary file 1 — Supplementary Material 1 [file 12870_2023_4513_MOESM1_ESM.docx]

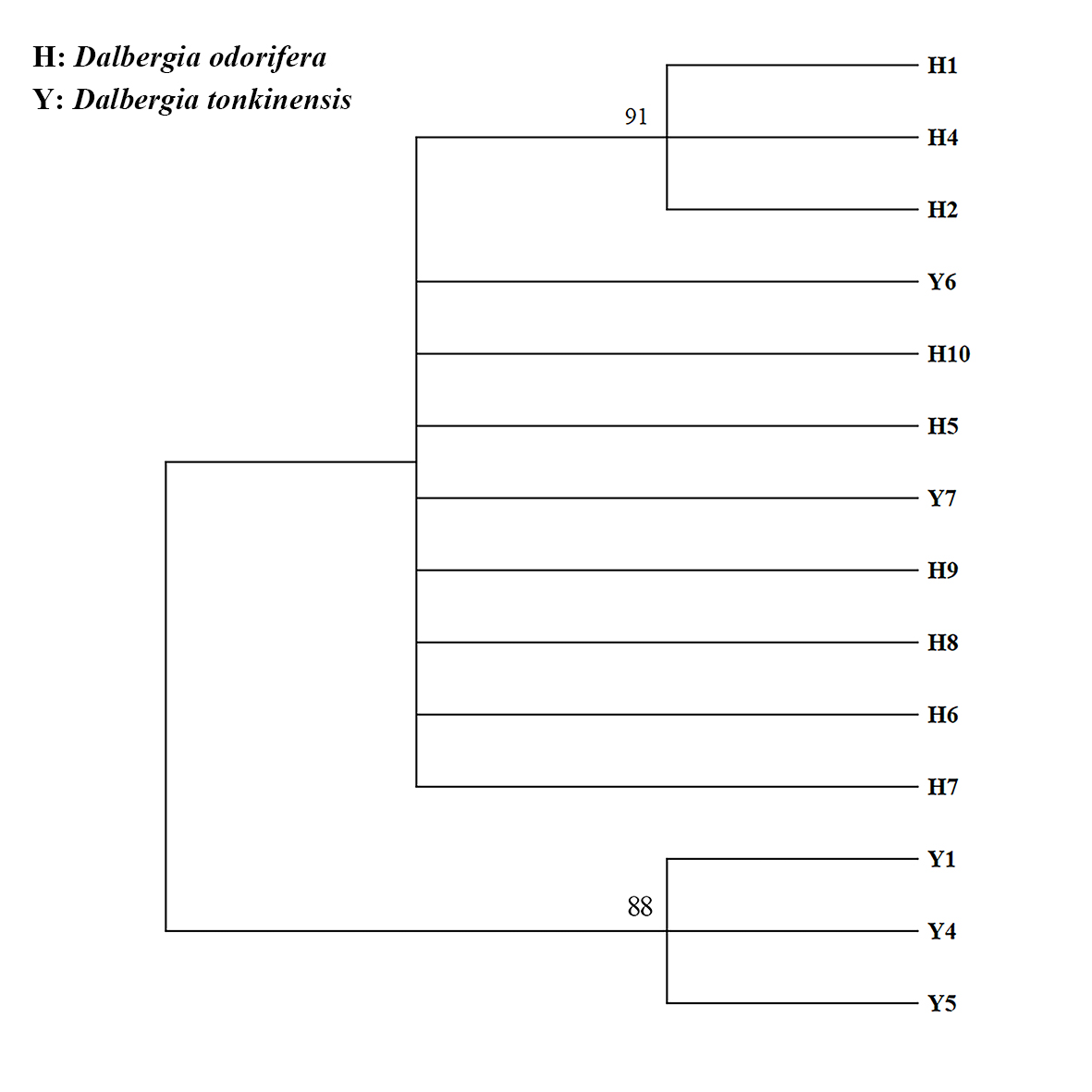


Fig. S1 NJ tree constructed based on *trnL-trnF*


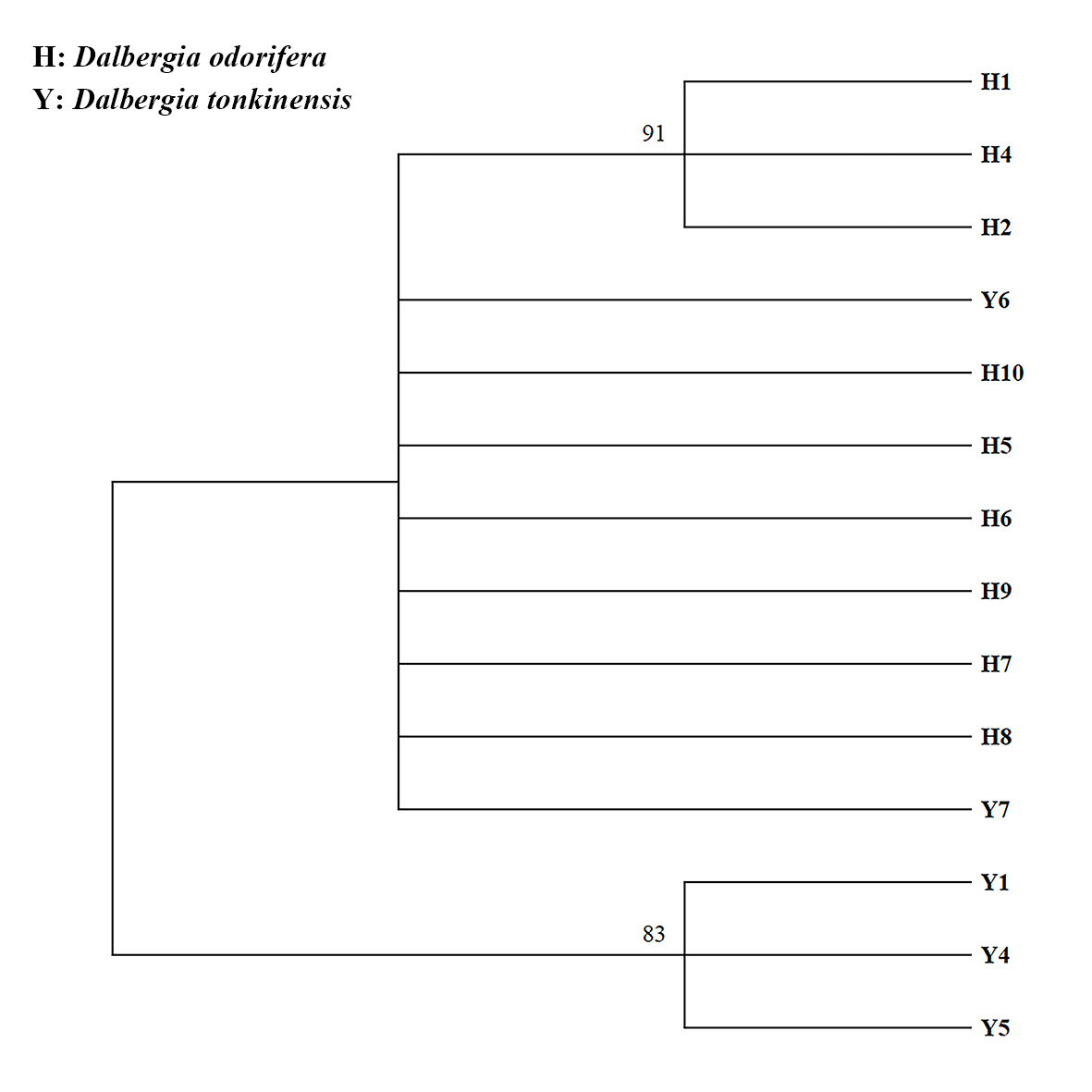


Fig. S2 NJ tree constructed based on *trnL-trnF*+ITS2


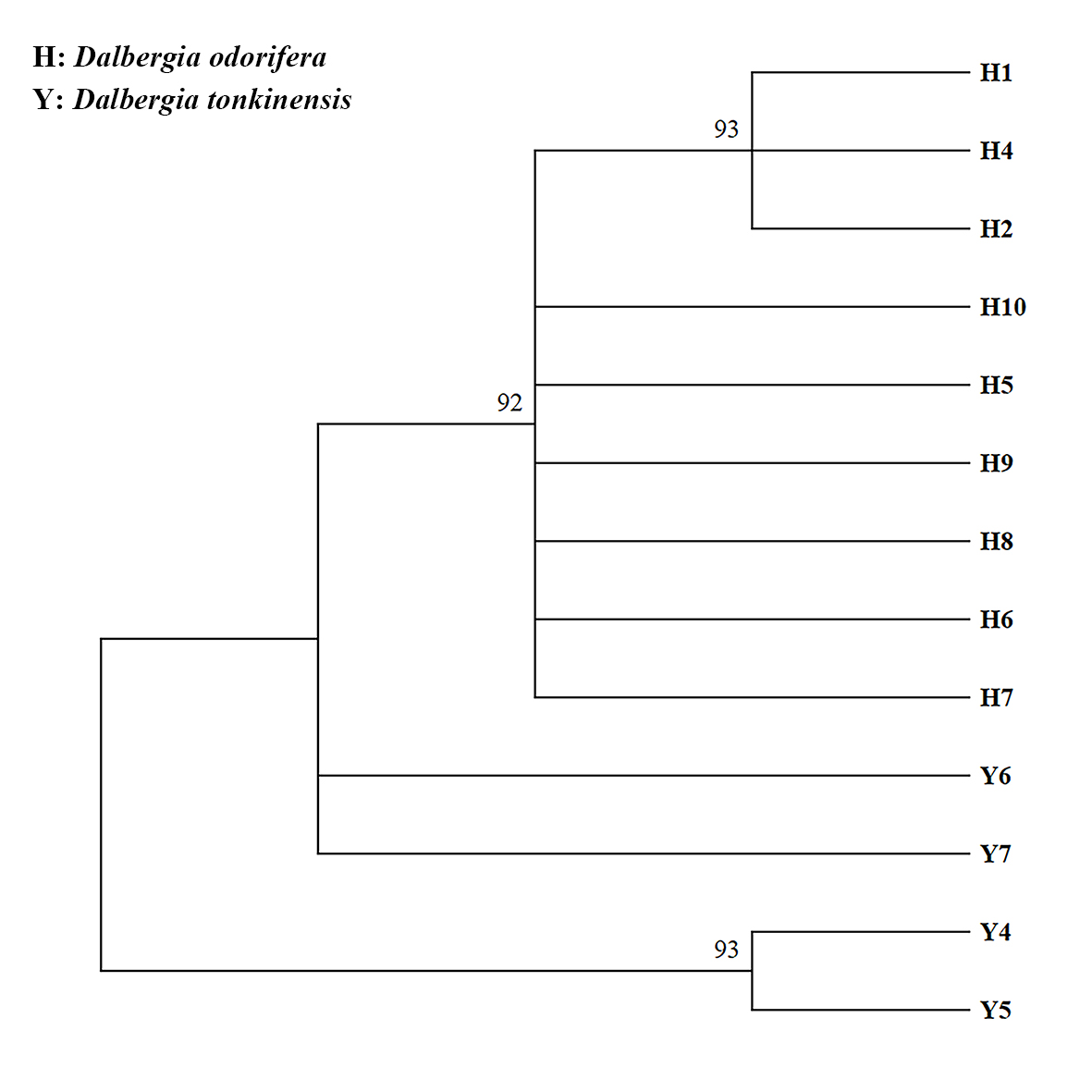


Fig. S3 NJ tree constructed based on *trnL-trnF*+*trnH-psbA*


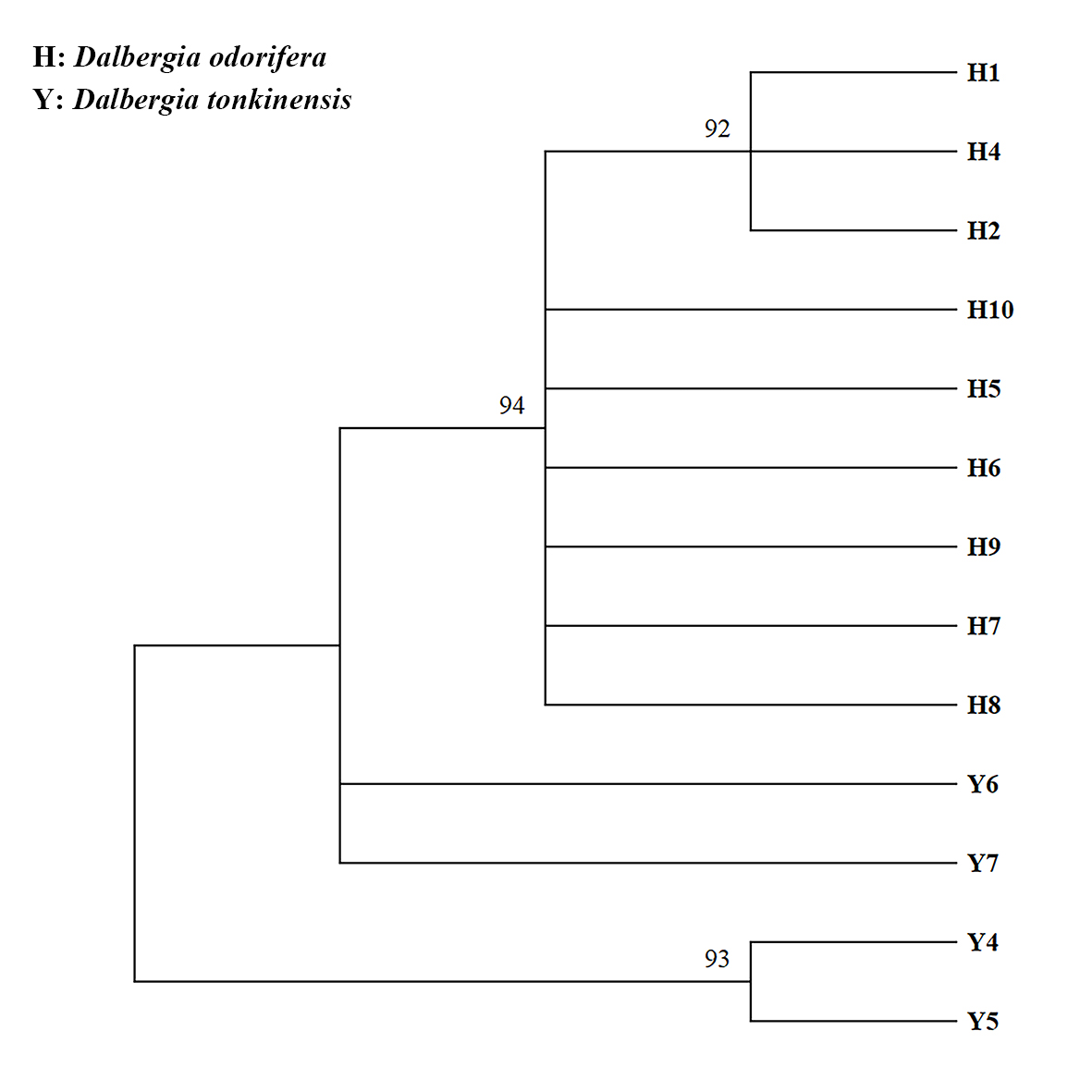


Fig. S4 NJ tree constructed based on *trnL-trnF*+*trnH-psbA*+ITS2
