# Supplementary material for: The DNA barcode identification of Dalbergia odorifera T. Chen and Dalbergia tonkinensis Prain
Source: BMC Plant Biol. 2023 Nov 7;23:546. doi: 10.1186/s12870-023-04513-3 (PMC10629101; doi:10.1186/s12870-023-04513-3)
Supplement: Supplementary file 3 — Supplementary Material 3 [file 12870_2023_4513_MOESM3_ESM.docx]

Table S2 Sequences and primers information

| No. | Sequences | Primers (3’-5’) | References |
| --- | --- | --- | --- |
| 1 | ITS2 | F:ATGCGATACTTGGTGTGAAT R:GACGCTTCTCCAGACTACAAT | [1] |
| 2 | *rpoB* | F:ATGCAACGTCAAGCAGTTCC R:GATCCCAGCATCACAATTCC | [2] |
| 3 | *rpoC1* | F:GTGGATACACTTCTTGATAATGG R:TGAGAAAACATAAGTAAACGGGC | [2] |
| 4 | *trnH-psbA* | F:ACTGCCTTGATCCACTTGGC R:CGAAGCTCCATCTACAAATGG | [2] |
| 5 | *trnL-trnF* | F:GGTTCAAGTCCCTCTATCCC R:TTTGAACTGGTACACGAG | [3] |
| 6 | *matK* | F:CGATCTATTCATTCAATATTTC R:TCTAGCACGAAAGTCGAAGT | [4] |
| 7 | *ycf3* | F:AGAACCGTACTTGAGAGTTTCC R:CTGTCATTACGTGCG(A/G)CTATCT | [5] |
| 8 | *trnL* intron | F:CGAAATCGGTAGACGCTACG R:GGGGATAGAGGGACTTGAAC | [3] |
| 9 | *trnS-psbC* | F:GCAGCTGCAGCAGGATTTG R:GGAGAGATGGCCGAGTGGTT | [5] |
| 10 | *rbcL* | F:ATGTCACCACAAACAGAAAC R:TCGCATGTACCTGCAGTAGC | [6] |

References:

1. Jianping H, Chang L, Minhui L, Lin-chun S, Jingyuan S, Hui Y, Xiaohui P, Shilin CH. Relationship between DNA barcoding and chemical classification of *Salvia* medicinal herbs. Chinese Herb Med. 2010;2:16-29.

2. Kress W, Erickson D. A two-locus global DNA barcode for land plants: the coding *rbcL* Gene complements the non-coding *trnH-psbA* spacer region. Plos One. 2007;2:e508.

3. Wang Y, Zhang S, Cui T. The utility of *trnL* intron and *trnL-trnF* IGS in phylogenetic analysis of Magnoliaceae. Acta Bot Bor-Occid Sin. 2003;23(2):247-252.

4. Lahaye R, van der Bank M, Bogarin D, Warner J, Pupulin F, Gigot G, Maurin O, Duthoit S, Barraclough TG, Savolainen V. DNA barcoding the floras of biodiversity hotspots. Proc Natl Acad Sci. U.S.A. 2008;105(8):2923-2928.

5. Jiao L, Yin Y, Xiao F, Sun Q, Song K, Jiang X. Comparative analysis of two DNA extraction protocols from fresh and dried wood of *Cunninghamia lanceolata* (Taxodiaceae). IAWA J. 2012;33:441-456.

6. Li M, Cao H, BUT P, Shaw P. Identification of herbal medicinal materials using DNA barcodes. J Syst Evol. 2011; 49(3):271-283
